# Postoperative recurrence of epithelial ovarian cancer patients and chemoresistance related protein analyses

**DOI:** 10.1186/s13048-019-0499-z

**Published:** 2019-03-27

**Authors:** Zhengmao Zhang, Kaiyun Qin, Wenzhe Zhang, Botao Yang, Chen Zhao, Xiaojing Zhang, Fenghua Zhang, Lianmei Zhao, Baoen Shan

**Affiliations:** 1 Department of Gynecology, Fourth Hospital of Hebei Medical University, Shijiazhuang, 050011 China; 2 Department of Gynecology and Obstetrics, Hebei General Hospital, Shijiazhuang, 050000 China; 3 Department of Gynecology and General Medical Ward, Fourth Hospital of Hebei Medical University, Shijiazhuang, 050011 China; 4 Department of Gynecology and Obstetrics, Fourth Hospital of Shijiazhuang, Shijiazhuang, 050011 China; 5 Department of General Surgery, Hebei General Hospital, Shijiazhuang, 050000 China; 6 Research Center, Fourth Hospital of Hebei Medical University, No.12, Jiankang Road, Shijiazhuang, 050011 China

**Keywords:** Epithelial ovarian cancer, Chemotherapy resistance, Differentially expressed proteins, Two dimensional gelelectrophoresis, Time of flight mass spectrometry, Proteomics

## Abstract

**Objective:**

To identify the plasma protein biomarkers related to the chemoresistance of postoperative recurrence of epithelial ovarian cancers.

**Methods:**

Forty plasma samples from patients in chemotherapy-sensitive and chemotherapy-resistant groups (20 for each group) were collected at Gynecology Department in the Fourth Hospital of Hebei Medical University from September 2013 to September 2014. The differentially expressed proteins between two groups were screened with two-dimensional gel electrophoresis (2-DE) and further analyzed by matrix-assisted laser desorption/ionization time of flight mass spectrometry (MALDI-TOF–MS).

**Results:**

Thirty-four differentially expressed spots were identified between the two groups. Compared with the chemo-sensitive group, 21 protein spots were up-regulated and 13 were down-regulated in the chemoresistant group, in which 14 differentially expressed proteins were identified by the Mass spectrometry and Mascot search. Among the 14 proteins, complement C4-A, IgJ chain, clusterin, α-1-antitrypsin and carbonic anhydrase 1 were up-regulated, and transthyretin, haptoglobin, β-2-glycoprotein, Ig γ-2 chain C region, Ig γ-1 chain C region, complement factor I light chain, Igκ chain C region, complement C3 and apolipoprotein E were down-regulated in the chemoresistant group when compared with the chemosensitive group.

**Conclusion:**

The up-regulated proteins including transthyretin, apolipoprotein E and haptoglobin proteins and the down-regulated proteins such as clusterin, carbonic anhydrase 1, alpha-1-antitrypsin were differentially expressed in the plasma between the chemo-sensitive group and the chemoresistant group, which may be potential biomarkers for predicting the chemotresistance of epithelial ovarian cancer patients.

## Introduction

Ovarian cancer is one of the three major malignant tumors of female genitalia. Epithelial ovarian tumor is the most common ovarian cancer. Due to no obvious early symptoms, more than 70% of epithelial ovarian cancer patients are diagnosed at advanced stages [1]. Even received systematic treatments of surgery and chemotherapy, about 70% of ovarian cancer patients still undergo recurrence and tumor progression [2]. It has been reported that 1/4 of ovarian cancer patients have primary resistance to chemotherapy regimens and 4/5 of patients may develop secondary resistance following chemotherapy [3]. Although the mechanisms of tumor multidrug resistance with the advance of genomics and second-generation sequencing, especially RNA sequencing, have been investigated, the mechanisms of chemoresistance of ovarian cancer have not been fully understood [4].

Exploration into the mechanisms at the protein level may be helpful to elucidate the cancer drug resistance-related mechanism. Proteomics can identify all the proteins of cells, tissues or organisms, and analyze the function and pattern of proteins [5]. Analysis of serum samples from ovarian cancer patients and normal women by surface enhanced laser desorption/time-of-flight mass spectrometry (SELDI-TOF-MS) have identified three protein markers, combination of which with CA125 could effectively improve the diagnosis of ovarian cancer [6].

To find out the relationship of chemotherapy resistance related plasma protein markers and postoperative recurrence in patients with epithelial ovarian cancer, we used the 2D gel electrophoresis-mass spectrometry in combination with the patient’s postoperative recurrence within 6-month after 6–8 cycle chemotherapy completed, and performed comparative analysis of plasma protein expression profiles of epithelial ovarian cancer chemotherapy-sensitivity and chemotherapy-resistance.

## Materials and methods

### Patients

This study was approved by the Ethics Committee of Hebei Medical University and signed consent was obtained from each patient. Forty patients from September 2013 to September 2014 at Gynecology Department in The Fourth Hospital of Hebei Medical University were enrolled in the study. All patients were diagnosed to be epithelial ovarian cancer and underwent complete ovarian cancer excision or ovarian tumor cell subtraction surgery. The diagnoses were further verified at postoperative pathological confirmation. After surgery, all patients received 6–8 cycles of the standard of platinum-based chemotherapy, and were followed up to at least 6 months. The 6–8 cycles of treatment consisted of platinum-based combination chemotherapy with paclitaxel of 135-175 mg/m2, carboplatin AUC 5-7 mg/ml/min, oxaliplatin of 130 mg/m2, and nedaplatin of 80-100 mg/m2. Inclusion criteria are as following: Pathological types were epithelial ovarian cancer; there was no history of other malignant tumors; the initial treatment was paclitaxel and carboplatin-based chemotherapy of 6 to 8 courses with follow-up satisfactory cytoreductive surgery or comprehensive staging surgery for early-staged ovarian cancer. Exclusion criteria are as following: Non-epithelial ovarian cancer; in the absence of a comprehensive staging of early ovarian cancer, or satisfactory cytoreductive surgery or without completion of 6–8 courses of chemotherapy; with other malignancies or history other tumors.

### Classification criteria

According to the National Comprehensive Cancer Network (NCCN) guidelines, after the initial treatment of platinum-based chemotherapy, the cancer recurrence within 6 month was defined as ovarian cancer chemotherapy-resistant, while the recurrence with more than 6-month were defined as ovarian cancer chemotherapy- sensitive. Ovarian cancer clinic outcome can be classified into following types: 1) chemotherapy-sensitive: after the initial administration of platinum-based chemotherapy, the patient has reached clinical remission but undergoes recurrence after more than 6-month following the termination of chemotherapy; 2) chemotherapy resistant: after the initial administration of platinum-based chemotherapy, the patient has reached clinical remission but undergoes recurrence within 6-months following termination of chemotherapy; 3) persistent ovarian malignancy: after the initial administration of platinum-based chemotherapy, the patient has demonstrated reaction or response but shows tumors after further examination; 4) refractory ovarian malignancy: platinum-based chemotherapy is ineffective and the tumor undergoes stabilization or progression. The chemotherapy-resistant, persistent ovarian malignancy and refractory ovarian malignancies are classified in the ovarian cancer chemotherapy-resistant group.

### Reagents and instruments

Urea, thiourea, dithiothreitol (DTT), 3-[3-(cholamidopropyl) dimethylamino] propanesulfonic acid salt (CHAPS), iodoacetamide, tricarboxylaminomethane (Tris), glycine and low melting point agar (10 cm) were from GE Corporation (Shanghai, China); sugar, trichloroacetic acid (TCA), ammonium persulfate (APS), sodium carbonate, N, N, N ‘, N’-tetramethyldiethylamine (TEMED), silver nitrate sodium thiosulfate and EDTA were from Amresco (Washington DC, USA); DC protein assay kit was from Bio-Rad (CA, USA). Bovine serum albumin (BSA) was purchased from Pierce; albumin and IgG protein removal and desalting kits were Merck (NJ, USA); trypsin was from Promega (Madison, WI, USA); formic acid, acetonitrile, tris-HCl (pH 6.8, pH 8.5, pH 8.8), coomassie Brilliant blue R-250, coomassie brilliant blue G-250, bromophenol blue, acetone, methanol, phosphoric acid, n-butanol, ethanol, glacial acetic acid, ammonium sulfate were obtained from Sigma (St. Louis, MI, USA). Low temperature high speed centrifuger (TTICH) was from Heraeus (Hanau, Germany); ultraviolet spectrophotometer was from Beckman (Bernard, FL, USA), eppendorf refrigerated centrifuge was from Eppendorf (German); IPGhor isoelectric focusing instrument, DALT-SIX SDS (TTICH type) and ImageScanner Scanner were from Shanghai GE Healthcare; PDquest Analysis Software was from Bio-Rad; ABI 5800 MALDI-TOF/TOF Tandem Mass Spectrometer was from ABI (USA).

### Plasma samples

The morning fasting peripheral venous blood of 2 ml from each patient was placed into EDTA anticoagulation vacuum blood collection tube (EP tube) and centrifuged at 3000 rpm for 15 min. The plasma was aspirated and divided into 5 EP tubes with 150 μl per tube. The plasma was stored in a refrigerator at − 80 °C.

### Plasma pretreatment

The frozen samples were removed from − 80 °C and thawed. Plasma samples in the same group were mixed and the high abundant proteins in the samples were removed using the albumin and IgG protein removal kit. The salt ion was removed by a desalting kit and then the sample was added with protein lysis buffer containing 9 M of urea, 1% IPG buffer (GE Healthcare), 1% Dithiothreitol (DTT), 4% of 3- [3- (cholamidopropyl) dimethylamino] propanesulfonic acid salt (CHAPS). After incubation in 30 °C water bath for 1 h, the sample was centrifuged at 15000 g for 15 min at room temperature. The supernatant was aspirated and centrifuged again at 15000 g for 15 min at room temperature. The supernatant from the second centrifugation was taken and the protein concentration was determined with DC Protein Assay kit.

### 2D-gel electrophoresis and staining

A total of 300 μg protein sample was adjusted to a total volume of 450 μL with sample hydrating solution and loaded onto the slot of IPG strip (GE Healthcare, 24 cm, pH = 3–10). Isoelectrophoresis was performed at 20 °C with the maximum current of 50 μA/strip. The isoelectric focusing procedure was: 50 V for 12 h, 500 V for 1 h, 1000 V for 1 h, gradient from1000 to 10,000 V for 1 h, and finally 10,000 V for 10 h. After soaking in SDS-PAGE electrophoresis buffer for 10 min, the gel was transferred to an electrophoresis tank of Ettan-DALT-Six system and electrophoresed at 100 V for 45 min and then 200 V for bromine to run out of the gel. The gel was then subjected to silver nitrate staining as described [7].

### Gel scanning and image analysis

The images were scanned with the Image Scanner with the optical density of 300dpi. The images were analyzed by PDquest 8.0 software. The main operations included gel protein spot detection, image background subtraction, protein point gray value standardization, and different gel protein spots matching. The 2-D electrophoretic images were compared and the protein spots with 2 times or less than 0.5 times were selected as the differential protein spots. The differential protein spots were selected as the follow-up mass spectrometry.

### Protein identification by mass spectrometry

The protein was digested and extracted with 50 μL extraction buffer. The protein extract was lyophilized and the dry powder was redissolved with 5 μL of 0.1% TFA solution. The solution was mixed with 5 μL saturated α-cyano-4-hydroxycinnamic acid containing 50% ACN and 1% TFA. 1 μL of the sample was subjected to mass spectrometry (MALDI-TOF/TOF) using ABI5800 Series Time-of-Flight Mass Spectrometer. The data were automatically acquired using positive ion mode. The primary and secondary mass spectrometry data were integrated using GPS 3.6 (Applied Biosystems) and Mascot2.3 (Matrix Science) to analyze and identify proteins. The search parameters are as follows: the database is human database; the enzyme is Trypsin; the maximum allowable breakpoint is 1; the fixed modification is carbamidomethyl (C); the variable modification is Acetyl (Protein N-term), deamidated (NQ), dioxidation (W) and oxidation (M); MS tolerance is 100 ppm and MS/MS tolerance is 0.5 Da. Protein score C.I. % of greater than 95% is defined as successful identification of a protein.

### Statistical analysis

All data were analyzed using SPSS19.0 statistical software. Comparison was made by *t* test between two groups. A *P* value of < 0.05 was considered statistically significant.

## Results

**Fig. 1:**
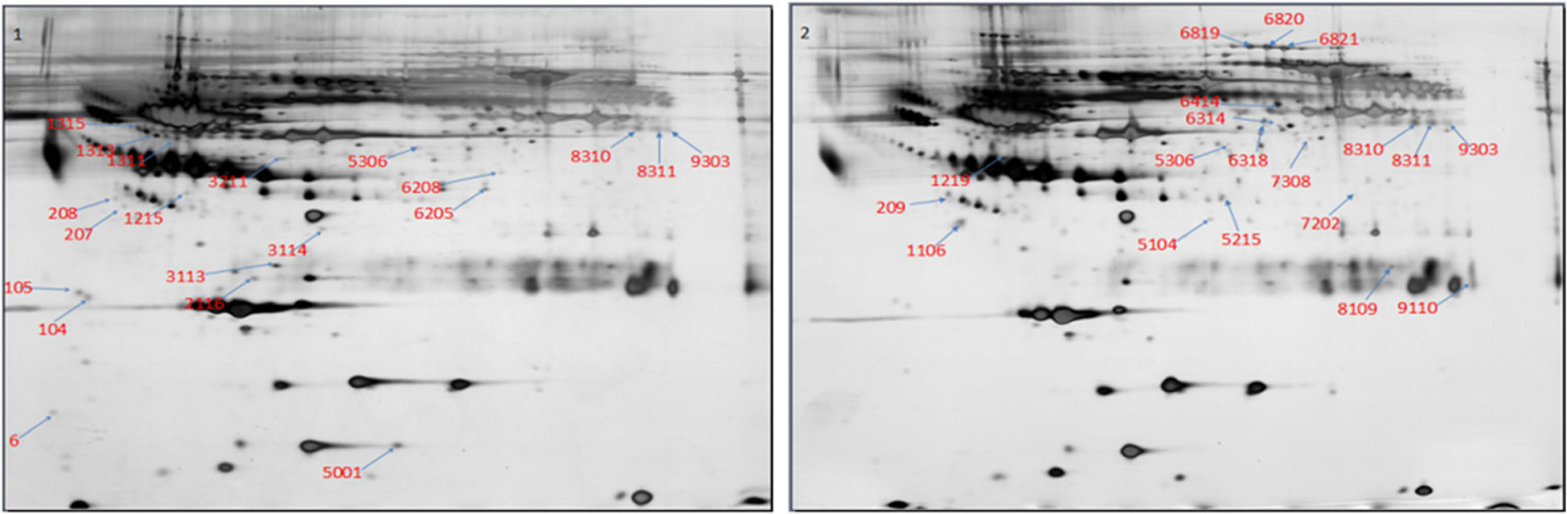
The significantly differentially stained protein spots in the 2-DE between the chemoresistant group and chemosensitive group. Reprehensive 2-DE silver staining was shown for the chemoresistant group (1) and chemosensitive group (2). The red number shows the spot identification number

The clinical characteristics of the study subjects are listed in Table 1. The plasma samples from postoperative epithelial ovarian cancer patients were in chemotherapy-sensitive and chemotherapy-resistant groups. Pooled plasma from 20 patients in each group was subjected into the two-dimensional gel electrophoresis (2-DE) and silver nitrate staining. The 2-DE was repeated for 3 times. After comparison of the 2-DE patterns between the two groups, 34 significantly altered spots were identified (Fig. 1). In comparison to that in chemotherapy-sensitive group, 21 spots were found significantly upregulated and 13 spots were found significantly downregulated in the chemotherapy-resistant group (Table 2).

**Table 1:** Clinical characteristics of the study subjects

No.	Age	FIGO stage	Histotype	Grade^A^	Regimen after primary surgery	Evaluation
1	61	III	Serous	III	Paclitaxel/ Oxaliplatin	Sensitive
2	44	III	Serous	II	Paclitaxel/Nedaplatin	Sensitive
3	60	II	Endometrioid	II	Paclitaxel/Nedaplatin	Sensitive
4	50	III	Serous	II	Paclitaxel/Oxaliplatin	Sensitive
5	55	III	Serous	II	Paclitaxel/Oxaliplatin	Sensitive
6	43	I	clear cell carcinomas	II	Paclitaxel/Oxaliplatin	Sensitive
7	51	III	Serous	II	Paclitaxel/Oxaliplatin	Sensitive
8	59	III	Endometrioid	II	Paclitaxel/Oxaliplatin	Sensitive
9	50	III	Serous	II	Paclitaxel/Oxaliplatin	Sensitive
10	55	II	Serous	III	Paclitaxel/Oxaliplatin	Sensitive
11	50	I	Serous	III	Paclitaxel/Oxaliplatin	Sensitive
12	56	III	Serous	III	Paclitaxel/Oxaliplatin	Sensitive
13	65	II	Endometrioid	III	Paclitaxel/Nedaplatin	Sensitive
14	58	II	Serous	III	Paclitaxel/Oxaliplatin	Sensitive
15	50	III	Serous	II	Paclitaxel/Oxaliplatin	Sensitive
16	59	III	Endometrioid	II	Paclitaxel/Nedaplatin	Sensitive
17	60	III	Serous	II	Paclitaxel/Oxaliplatin	Sensitive
18	63	III	Serous	II	Paclitaxel/Oxaliplatin	Sensitive
19	67	III	Serous	II	Paclitaxel/Oxaliplatin	Sensitive
20	58	III	Serous	II	Paclitaxel/Oxaliplatin	Sensitive
21	58	III	Serous	III	Paclitaxel/Oxaliplatin	Resistance
22	56	III	Serous	II	Paclitaxel/Oxaliplatin	Resistance
23	63	III	Serous	II	Paclitaxel/Oxaliplatin	Resistance
24	63	III	Serous	III	Paclitaxel/Nedaplatin	Resistance
25	68	IV	Serous	II	Paclitaxel/ Carboplatin	Resistance
26	50	III	Serous	III	Paclitaxel/Oxaliplatin	Resistance
27	48	III	Serous	III	Paclitaxel / Carboplatin	Resistance
28	49	III	Serous	III	Paclitaxel/ Nedaplatin	Resistance
29	63	IV	Serous	II	Paclitaxel/ Nedaplatin	Resistance
30	56	III	Endometrioid	III	Paclitaxel/Oxaliplatin	Resistance
31	61	IV	Serous	II	Paclitaxel/Nedaplatin	Resistance
32	40	II	Serous	II	Paclitaxel/Nedaplatin	Resistance
33	50	III	Serous	II	Paclitaxel/Oxaliplatin	Resistance
34	65	III	Serous	II	Paclitaxel/Oxaliplatin	Resistance
35	59	III	Serous	II	Paclitaxel/Oxaliplatin	Resistance
36	40	IV	Serous	II	Paclitaxel/Oxaliplatin	Resistance
37	62	III	Serous	II	Paclitaxel/ Carboplatin	Resistance
38	46	III	Serous	II	Paclitaxel/Oxaliplatin	Resistance
39	52	III	Serous	II	Paclitaxel/Oxaliplatin	Resistance
40	53	III	Serous	II	Paclitaxel/Nedaplatin	Resistance

A is graded according to the degree of differentiation (I-low grade, II- middle grade, III- high grade)

**Table 2:** The significantly differentially stained spots in the 2-DE between chemoresitant and chemosensitive groups

SSP	Ratio (chemo-resistant group/ chemo-sensitive group)	*P*-value
6	2.0799	0.000555
104	2.7969	0.017509
105	3.4434	0.001989
207	2.5022	0.034636
208	3.9958	0.001097
209	2.2814	0.019464
1106	0.3029	0.012599
1215	0.0856	0.065846
1219	0.3007	0.017823
1311	4.7495	0.045387
1313	2.6520	0.003545
1315	2.0011	0.040735
2116	4.6247	0.026594
3113	5.0058	0.017535
3114	0.2419	0.028733
3211	3.1774	0.040411
5104	0.3317	0.065635
5215	9.7176	0.002081
5306	0.3802	0.013318
6205	9.4899	0.018572
6208	2.5295	0.008858
6314	0.2573	0.011767
6318	2.2818	0.014183
6414	0.2360	0.042952
6819	43.7485	0.009163
6820	6.7241	0.000850
6821	4.8284	0.005170
7202	2.9882	0.038438
7308	0.2662	0.010295
8109	2.2008	0.053481
8310	0.0045	0.006954
8311	0.0063	0.004300
9110	0.0363	0.012862
9303	0.0062	0.023083

**Fig. 2:**
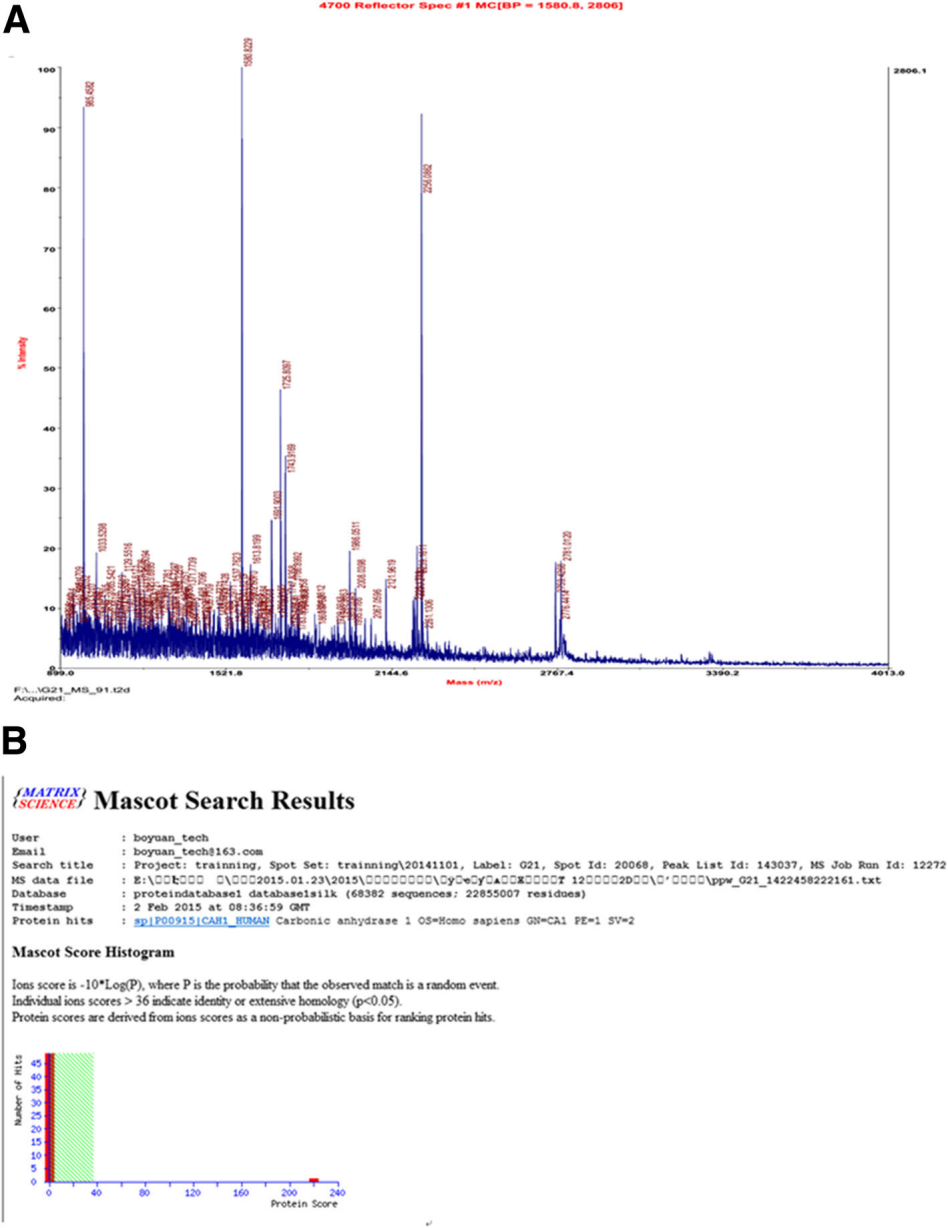
Identification of Carbonic anhydrase. **a**, mass spectrum. **b**, The Mascot analysis results of biological information

The differentially expressed 34 spots were subjected to mass spectrometry and Mascot2.3 analyses (Fig. 2). Fourteen differentially expressed proteins were identified between the two groups. In comparison that of the chemotherapy-sensitive group, Complement C4-A, IgJ chain, Clusterin, α-1-antitrypsin and Carbonic anhydrase 1 were upregulated, and Transthyretin, Haptoglobin, β-2-glycoprotein, Ig γ-2 chain C region, Ig γ-1 chain C region, Complement factor I light chain, Igκ chain C region, complement C3 and Apolipoprotein E were down-regulated in the chemotherapy-resistant group (Table 3).

**Table 3:** The identification of possible chemoresistant proteins in the plasma of patients with epithelial ovarian cancer recurrence by MALDI-TOF/TOF-MS

Spot No.	Protein name	Accession No.	MW/PI	Protein score
3113\6	Complement C4-A	GI:476007827	194,261/6.65	68
1106	Transthyretin	GI:37483	15,991/5.52	69
104\105	Immunoglobulin J chain	GI:21489959	18,543/5.12	83
209	Clusterin	GI:30251	53,031/5.89	119
9110	Ig kappa chain C region	GI:125145	11,773/5.58	44
1311	Alpha-1-antitrypsin	GI:50363217	46,878/5.37	34
1219	Complement C3	GI:115298678	188,569/6.02	45
5104	Haptoglobin	GI:386783	41,381/5.44	49
6414	Beta-2-glycoprotein	GI:28814	39,584/8.34	74
8311	Ig gamma-2 chain C region	GI:218512079	36,505/7.66	43
9303	Ig gamma-1 chain C region	GI:121039	36,596/8.46	51
8109	Carbonic anhydrase	GI:29600	28,909/6.59	220
1215	Complement factor I light chain	GI:119626656	67,411/7.72	73
3114	Apolipoprotein E	GI:114039	36,246/5.65	421

## Discussion

Ovarian cancer mortality rate ranks the first in gynecological malignancies and the 5-year survival rate of advanced ovarian cancer patients is 30–40% [8]. Resistance to chemotherapy drugs is one of the important factors affecting the prognosis of patients, however the underlying molecular mechanisms remain largely unclear [4, 8]. In the present study, we compared the differentially expressed proteins of the plasma between the chemotherapy-resistant and chemotherapy-sensitive epithelial ovarian cancer patients by 2-DE and revealed 34 significantly differential expression spots. Further mass spectrometry and Mascot search found 14 differentially expressed proteins in the plasma between chemotherapy-resistant and chemotherapy-sensitive epithelial ovarian cancer patients. We found that Transthyretin was downregulated while Clusterin was upregulated in the plasma of chemotherapy-resistant ovarian cancer patients. Our findings suggest that these identified proteins may be potential biomarkers of chemotherapy resistance of ovarian cancer.

Transthyretin (TTP) also called thyroxine protein and prealbumin (PA), is a tetramer of four identical subunits. TTP plays a main role in the transport of thyroxine and retinol, and its level can accurately reflect the status of body’s protein synthesis [9, 10]. In 2005, Kozak et al. found that TTP expression in ovarian cancer was significantly reduced using liquid chromatography-mass spectrometry, and the combination of TTP with transferrin, apolipoprotein AIm hemoglobin and CA125 significantly improved the diagnosis of early ovarian cancer [5]. Similar to the previous study with the ascites from 12 patients with chemotherapy-sensitive and 7 patients with chemotherapy-resistant epithelial ovarian cancer [11], we found that the expression level of TTP in the plasma of ovarian cancer patients with chemoresistant was significantly lower than that of chemosensitivity patients. Thus, TTP may play an important role in chemotherapy resistance of ovarian cancer.

Clusterin is a heterodimer of sulfated glycoprotein abundantly found in body fluids and tissues and plays a critical role in cell aggregation [12], complement inhibition/regulation of immune [7], tissue repair, lipids transport and reproduction [13, 14]. In 1995, Lee et al. [6] first found that an anti-apoptotic effect of clusterin in prostate cancer cells and speculated a protective function of cluserin. After comparing CLUSTERIN gene overexpression and silencing in ovarian cancer cells, Wei et al. [15] revealed that nuclear Clusterin plays a role in promoting cell apoptosis, whereas secreted Clusterin protects cells. Increased expression of secreted Clusterin not only inhibited cell apoptosis and induced chemotherapy resistance, but also promoted the deterioration and progression of ovarian cancer cells [16, 17]. This coincides with our observation that Clusterin was significantly increased in the plasma of ovarian cancer patients with chemotherapy-resistant. These data suggest that Clusterin is a biomarker for predicting chemotherapy of ovarian cancer.

The present study identified several complement system proteins, including complement C4-A, complement C3, and complement factor I light chain. The complement C3a and C4a has a anaphylatoxins role. Complement C4 is a key factor in the classical complement pathway and is often up-regulated in autoimmune diseases. Serum complement system contains more than 30 proteins. Activation of complement system through three ways regulates signal transmission, immune, inflammation and other functions. In recent years, some proteomics studies have also found that some serum complement proteins may be associated with bladder cancer [18], breast cancer [19], and ovarian cancer [20]. The mechanism is more complex and not yet clear.

The development of malignant tumors is related to the immune function of the body, especially the cellular immune function [21], but little is known about humoral immunity. IgG is an immune antibody and has anti-toxin, anti-bacterial, anti-virus and allergy regulation functions. IgG is the most abundant serum immunoglobulin in normal human and plays an important role in specific immunity. Studies have reported that ovarian cancer prognosis and chemosensitivity are associated with the presence of immune cells and immune factors, but the majority of reports are related to T cells. The presence of T cells in ovarian cancer patients may predict good prognosis or chemosensitivity [22], whereas inhibition of T-Regs cells is usually associated with shorter survival [23]. Zhang Z et al. [24] reported that the positive rate of CD45RA expression in early stage epithelial ovarian cancer patients was significantly higher than that in advanced stage patients, and CD45RO, pathological grade and surgical stage affected the prognosis of epithelial ovarian cancer. Moreover, CD45RO + T lymphocytes and dendritic cells CD1a + or S-100 + is associated with a higher survival rate in patients with epithelial ovarian cancer.

In conclusion, by screening, identifying and analyzing with two-dimensional gel electrophoresis-mass spectrometry techniques, we revealed 14 differentially expressed proteins in the plasma between chemoresistant and chemosensitive patients with ovarian cancer. Our findings may provide the basis for further studies to predict chemotherapy resistance, to develop individualized treatment programs and to find ways to reverse chemotherapy resistance of ovarian cancer patients. However, a multi-centered study with a large cohort of patients is needed in the future to validate our findings.
